# A Machine Learning Approach to Identify Predictors of Potentially Inappropriate Non-Steroidal Anti-Inflammatory Drugs (NSAIDs) Use in Older Adults with Osteoarthritis

**DOI:** 10.3390/ijerph18010155

**Published:** 2020-12-28

**Authors:** Jayeshkumar Patel, Amit Ladani, Nethra Sambamoorthi, Traci LeMasters, Nilanjana Dwibedi, Usha Sambamoorthi

**Affiliations:** 1 Department of Pharmaceutical Systems and Policy, West Virginia University, Morgantown, WV 26506, USA; tlemasters@hsc.wvu.edu (T.L.); nidwibedi@hsc.wvu.edu (N.D.); usambamoorthi@hsc.wvu.edu (U.S.); 2 Rheumatology, West Virginia University Medicine, Morgantown, WV 26506, USA; amit.ladani@hsc.wvu.edu; 3 Masters in Data Science Program, School of Professional Studies, Northwestern University, Chicago, IL 60201, USA; nsm@crmportals.com; 4 Department of Pharmacotherapy, HSC College of Pharmacy, The University of North Texas Health Science Center at Fort Worth, Fort Worth, TX 76107, USA

**Keywords:** osteoarthritis, anti-inflammatory agents, non-steroidal, machine learning, cohort studies, aged, adverse drug event

## Abstract

Evidence from some studies suggest that osteoarthritis (OA) patients are often prescribed non-steroidal anti-inflammatory drugs (NSAIDs) that are not in accordance with their cardiovascular (CV) or gastrointestinal (GI) risk profiles. However, no such study has been carried out in the United States. Therefore, we sought to examine the prevalence and predictors of potentially inappropriate NSAIDs use in older adults (age > 65) with OA using machine learning with real-world data from Optum De-identified Clinformatics^®^ Data Mart. We identified a retrospective cohort of eligible individuals using data from 2015 (baseline) and 2016 (follow-up). Potentially inappropriate NSAIDs use was identified using the type (COX-2 selective vs. non-selective) and length of NSAIDs use and an individual’s CV and GI risk. Predictors of potentially inappropriate NSAIDs use were identified using eXtreme Gradient Boosting. Our study cohort comprised of 44,990 individuals (mean age 75.9 years). We found that 12.8% individuals had potentially inappropriate NSAIDs use, but the rate was disproportionately higher (44.5%) in individuals at low CV/high GI risk. Longer duration of NSAIDs use during baseline (AOR 1.02; 95% CI:1.02–1.02 for both non-selective and selective NSAIDs) was associated with a higher risk of potentially inappropriate NSAIDs use. Additionally, individuals with low CV/high GI (AOR 1.34; 95% CI:1.20–1.50) and high CV/low GI risk (AOR 1.61; 95% CI:1.34–1.93) were also more likely to have potentially inappropriate NSAIDs use. Heightened surveillance of older adults with OA requiring NSAIDs is warranted.

## 1. Introduction

Each year, nearly 29 million people in the United States use non-steroidal anti-inflammatory drugs (NSAIDs) for the relief of various types of pain [1]. These drugs are moderately effective in treating pain and are recommended by numerous clinical guidelines for its treatment [2,3]. However, their use is associated with several cardiovascular (CV) and gastrointestinal (GI) adverse events (AE), some of which can be life-threatening [4]. It is estimated that each year more than 100,000 adults are hospitalized and over 16,000 die from NSAIDs-related AEs in the USA [5,6]. On the basis of NSAIDs’ affinity to inhibit cyclooxygenase-2 (COX-2) enzyme, they can be classified as non-selective or selective drugs, which differ in their CV and GI safety profiles [4]. While there is a lower risk of GI AEs with selective NSAIDs, both non-selective and selective NSAIDs have a similar risk of CV AEs, except for naproxen and ibuprofen [4,7]. Research suggests that patients can experience NSAIDs-related AEs as early as four weeks after initiation [8,9]. Therefore, several clinical guidelines recommend that NSAIDs should be used at the lowest possible dose for the shortest duration [3,10]. Furthermore, it is also recommended that their use should be in accordance with a person’s CV or GI risk profile (i.e., appropriate NSAIDs use) to reduce the risk of NSAIDs-related AEs [3,10].

The examination of potentially inappropriate NSAIDs use among older individuals with osteoarthritis (OA) is particularly important for several reasons. First, OA is a progressive degenerative disease affecting as many as 30 million adults in the USA with disproportionately higher prevalence among older adults [11]. Second, pain is the defining feature of OA with one in four OA patients reporting some level of pain [12], and approximately 40% reporting moderate to severe pain interference with daily activities [13]. Therefore, it is no surprise that in real-world practice, around half of older adults with OA receive prescription NSAIDs for pain relief [1]. Third, the risk of NSAIDs-related AEs may be higher in older adults, due to age-related loss of physiological organ reserve, higher comorbidity burden, and polypharmacy [14]. Previous studies have estimated that NSAIDs use results in 41,000 hospitalizations and 3300 deaths per year among older adults [15]. Additionally, nearly one quarter (23.5%) of adverse drug reaction-related hospitalizations in older adults have also been found to be associated with NSAIDs [16]. Furthermore, in two previous studies [17,18], nearly half of older adults with OA were found to have CV and GI risk profiles unfavorable with NSAIDs use. The study by Vanderstraeten et al. evaluated the GI risk profiles of adult OA patients who were potential NSAID users [17]. On the basis of the data collected from 885 OA patients from Belgium and Luxemburg, the authors observed a higher prevalence of high GI risk (77.8% vs. 19.4% moderate or 2.8% low GI risk). The prevalence of high GI risk was even higher among older adults (86.4% vs. 70.7%) compared to their younger counterparts. Similarly, in a study comprising a majority of older adults with OA from clinics in Spain, 60.3% and 31.5% of OA patients were found to have high GI risk and high CV risk, respectively [18]. Additionally, 51% of those patients with unfavorable CV and GI risk profiles were found to be treated with potentially inappropriate NSAIDs. Given strict controls on opioid prescriptions, due to the opioid epidemic, older adults with OA may increasingly rely on prescription NSAIDs for pain relief. This may further lead to higher rates of potentially inappropriate NSAIDs use in this patient population.

Despite its importance, the research examining the potentially inappropriate NSAIDs use among older adults with OA is lacking in the USA. Studies conducted in other countries suggest that approximately half of adults with OA are not treated with NSAIDs appropriate with their GI and CV risk profiles [18,19]. Such potentially inappropriate use of NSAIDs can add to the already high burden of OA and may outweigh any potential benefits obtained from their use. It is important to identify the prevalence of potentially inappropriate NSAIDs use and its predictors to inform policy making, clinical practice, and to develop tailored interventions targeting older adults with OA who are already at a higher risk for CV and GI AEs.

Therefore, the primary objective of the current study is to examine the prevalence and leading predictors of potentially inappropriate NSAIDs use among older adults with OA using real-world data from nationally representative commercial health insurance claims with the help of machine learning approaches. We also examine the association of CV and GI risk profiles at baseline to potentially inappropriate NSAIDs use during the follow-up period.

## 2. Materials and Methods

### 2.1. Data Source

The study cohort was selected from a 10% random sample of all individuals in the Optum De-identified Clinformatics^®^ Data Mart (Optum) [20]. The deidentified Optum data contain commercial and Medicare Advantage claims from eligible enrollees. The data are geographically representative of the commercially insured USA population. This dataset provides comprehensive longitudinal data on demographics, diagnoses, medications, and other medical services provided to insured individuals. We used insurance claims from inpatient, outpatient, and pharmacy claims from the years 2015 and 2016 for the present study. As data are deidentified, informed consent from patients was not required. International Classification of Diseases, 10th Revision (ICD-10) codes, National Drug Codes (NDC), revenue codes, and Healthcare Common Procedure Coding System (HCPCS) codes were used to identify relevant clinical conditions, medications, and services and procedures.

### 2.2. Study Design

A retrospective cohort study design with the 2015 calendar year as the baseline and 2016 as follow-up was used. The cohort comprised of older adults (age ≥ 65 years) with any type of OA (hip, knee, and others) at baseline. CV and GI risk profiles, demographic characteristics, concurrent use of other potential OA treatments including intra-articular corticosteroid or hyaluronic acid injections, opioids, and physical therapy were identified during the baseline period. Data from the follow-up year were used to identify the type (COX-2 selective or non-selective), duration, and appropriateness of NSAIDs use.

### 2.3. Study Cohort

Older adults with OA were identified using one inpatient or two outpatient claims at least 30 days apart that consisted of OA diagnosis codes (ICD-10 codes M15–M19) during the baseline year [21]. We required that these adults be enrolled in Medicare Advantage plans with medical and pharmacy benefits during 2015 and 2016 (i.e., 24 months).

### 2.4. Measures

#### 2.4.1. Target (i.e., Dependent) Variable: Potentially Inappropriate NSAIDs Use during Follow-Up (Yes/No)

Although clinical guidelines recommend NSAIDs use for the “shortest” possible duration, there is no clear guidance on what duration is considered appropriate, especially among subgroups of patients like older adults who are at high risk for NSAIDs-related AEs. Therefore, we utilized expert clinical opinion, clinical evidence [4], practical guidelines [22,23], and published literature [24,25] to define potentially inappropriate duration of NSAIDs use. To define such use, we considered two factors: (1) CV and GI risk (see below), and (2) class of NSAIDs used (COX-2 selective or non-selective). Individuals without CV or GI risk (low CV/low GI risk) were classified as having potentially inappropriate NSAIDs use if they received any type (COX-2 selective or non-selective) of NSAIDs for 90 consecutive days, with an allowed gap of 7 days between prescription refills, due to advanced age. Individuals with GI or CV risk (see “Features” section for definitions) were considered to have potentially inappropriate NSAIDs use if they met any of the following criteria: (1) high CV/high GI risk and any type of NSAIDs use for 60 consecutive days or longer; or (2) high CV/low GI risk with selective or non-selective (except naproxen below 1000 mg/day or ibuprofen below 1200 mg/day) NSAIDs use for 60 consecutive days; or (3) low CV/high GI risk with non-selective NSAIDs use for 60 consecutive days or longer. This classification took into account the fact that there is a high risk of GI AEs with non-selective NSAIDs and a high risk of CV AEs with both non-selective and COX-2 selective NSAIDs except naproxen and ibuprofen up to certain dose. The lack of data on CV and GI risk factors from a year prior to the baseline year precluded the possibility to identify and exclude inappropriate NSAID users at baseline.

#### 2.4.2. Features (i.e., Independent Variables)

##### CV and GI Risk Categories for Older Adults

Older Adults with OA were classified into the following CV and GI risk categories: (1) high CV/high GI; (2) high CV/low GI; (3) low CV/high GI; and (4) low CV/low GI (See Table 1 for definitions). This classification was based on published guidelines or recommendations from the American College of Gastroenterology [26], American Heart Association [27], American College of Rheumatology [23], the National Institute for Health and Care Excellence [7], and expert opinion from our study clinician. Clinical guidelines often define patients with high GI risk as those having two or more minor GI risk factors listed on the left below, which excludes old age. Please note that as older age itself is a minor risk factor [28,29], and as our study sample included only older adults, the presence of at least one other minor GI risk factor placed a person in the high GI risk category. Similarly, a person with at least one of the CV risk factors mentioned in Table 1 was identified as having high CV risk. We used ICD-10 codes to identify CV and GI conditions (see Table S1 in supplementary materials) and NDC codes to identify concurrent medications from pharmacy claims during the baseline.

##### Other Features

All other features (including 31 features with 36 indicators) included in our analyses were guided by the Andersen’s Healthcare Utilization Model and previously published literature [30]. These features included: (1) predisposing factors: age and sex, (2) enabling: type of health insurance coverage, a fragmentation of care index (FCI) [31], pain-related treatment (opioid, intra-articular hyaluronic acid and corticosteroids, physical therapy); (3) need factors: chronic conditions including asthma, cancer, cardiac arrhythmias, chronic obstructive pulmonary disease (COPD), dementia, anxiety, depression, diabetes, hyperlipidemia, hypertension, substance abuse disorders, other pain conditions (e.g., arthritis and joint pain conditions other than osteoarthritis, headache, migraine, back or neck pain, and neuropathic pain conditions), pain related to OA (i.e., pain in joints commonly affected by OA including knee and hip), and duration and type of NSAIDs use (COX-2 selective vs. non-selective), (4) life-style practices (obesity), and (5) external environment (region). The FCI was calculated on the basis of a modified version of a previously validated continuity of care index [31]. The value of the FCI takes into account the following three factors: the total number of healthcare encounters, the number of different providers, and the proportion of encounters to each of the providers. The FCI ranges from 0 (all encounters with the same provider) to 1 (each encounter with a different provider) and higher values indicate fragmented care or care discontinuation. Higher values of the FCI may also indicate an unmet need and a higher severity of disease.

### 2.5. Analytic Approach

We chose two machine learning algorithms, namely cross-validated logistic regression (CVLR) and eXtreme Gradient Boosting (XGBoost), to identify the leading predictors of potentially inappropriate NSAIDs use as well as to examine the association of CV and GI risk factors to potentially inappropriate NSAIDs use. We selected these machine learning algorithms for their better predictive accuracy and their ability to handle outliers, multicollinearity, and high-level interaction terms [32].

Random forest and XGBoost are two of the most popular ensemble learning techniques that combine several machine learning techniques (e.g., bagging and boosting) to decrease variance, bias, and improve predictions [33]. Although both algorithms use multiple decision trees, the XGBoost utilizes gradient boosting, wherein it builds one tree at a time and takes into account the errors made by all previously built trees [34]. XGBoost has been shown to achieve excellent prediction accuracies in many classification tasks and is known for faster processing speeds and regularization to prevent overfitting [35].

In our study, data analyses involved the following steps: data pre-processing, model building, model validation, model performance evaluation, feature importance derivation, and interpretation of results (i.e., a positive or negative relationship of each variable with the target variable).

All data pre-processing tasks were performed using SAS 9.4 (SAS Institute Inc. 2010, Cary, NC, USA). In preparation for the XGBoost, we used one-hot encoding, a process in which the values of categorical variables were converted into binary indicators with values of zero and one.

We followed accepted best practices for machine learning while building the XGBoost and CVLR models. These included: a random split of our dataset into training (70%) and testing (30%) subsets; using the test (unseen) subset data to test the performance of the models; k-fold cross-validation to avoid overfitting; and tuning of hyper-parameters to achieve the best performance. We tuned XGBoost hyperparameters, which are configurations external to the model and whose values cannot be estimated from the data, during model training to achieve better performance and avoid overfitting. We used the grid search method of the caret package in R software suite version 3.6.3 (R Development Core Team; Vienna, Austria) for this task. The final set of hyperparameters used in our model included the number of trees (800), learning rate (eta = 0.01), minimal loss to expand on a leaf node (gamma = 0), maximum tree depth (max_depth = 4), subsample proportion (subsample = 0.75), proportion of variables to randomly pick from for each tree (colsample_bytree = 0.6), and the minimum sum of instance weight needed in a child node (min_child_weight = 1).

We used 3-fold cross-validation to validate our models. Model fit was then evaluated using area under the receiver operating curve (AUROC) for test data. We also compared models on other performance metrics such as accuracy, positive predictive value (i.e., precision), sensitivity (i.e., recall), specificity, F1-score (i.e., harmonic mean of precision and recall), and kappa statistic.

To improve the interpretability of the results from XGBoost and to summarize the associations of selected features to potentially inappropriate NSAIDs use, we used a novel interpretable machine learning technique called SHapley Additive exPlanations (SHAP) [36]. SHAP provides not only feature importance but it also shows how much each feature contributes to the model prediction (global interpretability). With SHAP, feature contribution is derived by estimating the average marginal contribution of a specific feature to the model by using permutation and combinations of all features. We present feature importance and feature contribution through visual representation using SHAP summary and feature importance plots. Shapley values for XGBoost model were obtained using SHAPforXGBoost package in R version 3.6.3 (R Development Core Team; Vienna, Austria).

To facilitate the comparison of our results with the published literature, we also present adjusted odds ratios (AOR) and 95% confidence intervals (CI) from the CVLR. All data management and descriptive statistics were completed with SAS 9.4. Machine learning algorithms were run using R version 3.6.3.

## 3. Results

### 3.1. Sample Characteristics

Our study cohort comprised of 44,990 older adults with OA with a mean age of 75.9 years, 66.4% were females, and 55.3% had OA-related pain during the baseline period (Table 2). Overall, a majority (56.1%) of our study cohort did not have CV or GI risk factors during the baseline period while 43.9% had at least one CV or GI risk factor. Specifically, 7.5% of older adults had high CV/high GI risk; 9.4% had high CV/low GI risk; and 27.0% low CV/high GI risk. Nearly 1 in 8 older adults (12.8%, *n* = 5772) had potentially inappropriate NSAIDs use during the follow-up period (Table 2).

### 3.2. Prevalence of Potentially Inappropriate NSAIDs Use

**Figure 1:**
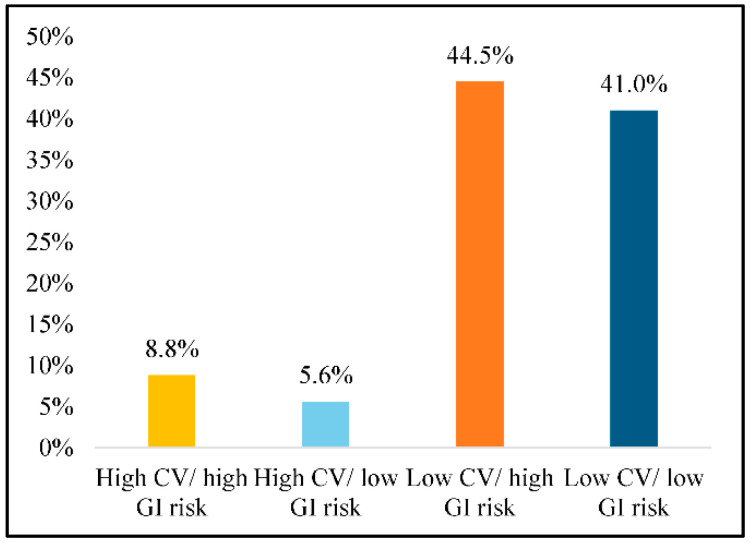
Percentage of Older Adults with Osteoarthritis and Potentially Inappropriate NSAIDs Use by Risk Categories. Note: Based on 44,990 older adults (age > 65) with OA using data from Optum De-identified Clinformatics^®^ Data Mart who were continuously enrolled in Medicare Advantage plan during 2015–2016. CV—cardiovascular; GI—gastrointestinal; NSAIDs—non-steroidal anti-inflammatory drugs; OA—osteoarthritis.

Figure 1 displays the percentage of our study cohort with potentially inappropriate NSAIDs use by CV and GI risk categories. The rates of potentially inappropriate NSAIDs use during the follow-up period were: 8.8% among older adults with high CV/high GI; 5.6% among those with high CV/low GI; 44.5% among those with low CV/high GI; and 41.0% among those with low CV/low GI.

### 3.3. Unadjusted Associations of Baseline Characteristics and Potentially Inappropriate NSAIDs Use

**Figure 2:**
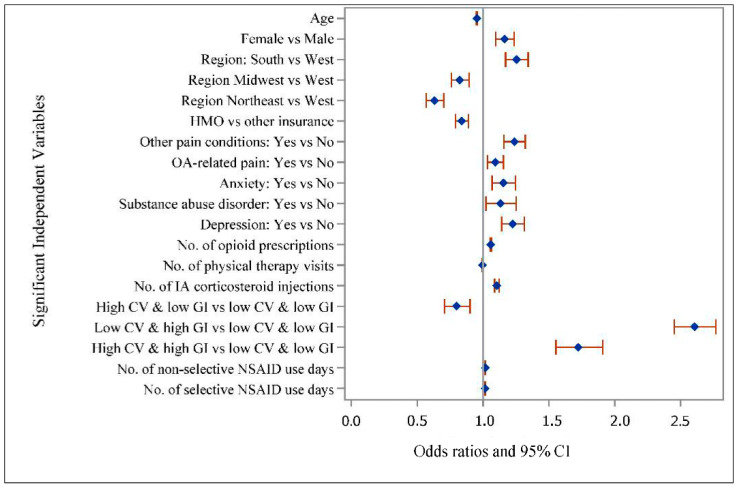
Unadjusted Odds Ratios and 95% CI for Potentially Inappropriate NSAIDs Use in Older Adults with Osteoarthritis. Note: Based on 44,990 older adults (age > 65) with OA using data from Optum De-identified Clinformatics^®^ Data Mart who were continuously enrolled in Medicare Advantage plan during 2015–2016. Unadjusted associations significant at *p* < 0.05. CV—cardiovascular; GI—gastrointestinal; HMO—health maintenance organization; IA—intra-articular; NSAIDs—non-steroidal anti-inflammatory drugs; OA—osteoarthritis.

Figure 2 presents the unadjusted odds ratios (UOR) and 95% CIs for potentially inappropriate NSAIDs use by selected baseline characteristics. Compared to the older adults with low CV/low GI risk, those with low CV/high GI (UOR: 2.61; 95% CI:2.45–2.77) and high CV/high GI (UOR: 1.72; 95% CI:1.55–1.91) were more likely to have potentially inappropriate NSAIDs use and those with high CV/low GI (UOR: 0.80; 95% CI:0.71–0.90) were less likely to have potentially inappropriate NSAIDs use. With respect to other factors, higher age and enrollment in an health maintenance organization (HMO) plan were associated with lower odds of potentially inappropriate NSAIDs use. On the other hand, female gender, other pain conditions, OA-related pain, anxiety disorders, substance abuse disorder, depression, and a higher number of opioid prescriptions or intra-articular corticosteroid injections were associated with higher odds of potentially inappropriate NSAIDs use.

### 3.4. Model Performance Comparison

Both XGBoost and CVLR were trained on a set of 31 features. Performance metrics for models using test data are presented in Table 3. Both models had an AUROC value of 0.92 (95% CI: 0.91–0.93) and 0.91 (95% CI: 0.90–0.92), respectively. While both models had similar accuracy and specificity, CVLR had better precision (0.83 vs. 0.81). On the other hand, XGBoost performed better on all other metrics being compared, including recall, F1 score, and kappa statistic.

### 3.5. Feature Importance and Model Interpretation

**Figure 3:**
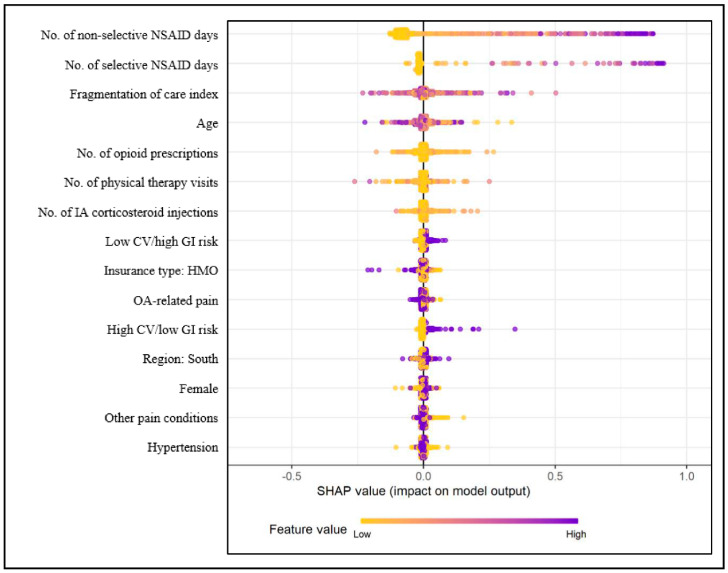
Variable Importance and SHAP Summary Plot for Potentially Inappropriate NSAIDs Use. Note: Based on 44,990 older adults (age > 65) with OA using data from Optum De-identified Clinformatics^®^ Data Mart who were continuously enrolled in Medicare Advantage plan during 2015–2016. The x-axis represents the marginal contribution of a feature to the change in predicted probability of NSAIDs use. Feature value refers to the actual value of the predictors (e.g., values for no. of non-selective NSAIDs days will range from 0 to 365 with values closer to 0 represented with yellow and values closer to 365 represented with purple dots). CV—cardiovascular, GI—gastrointestinal, HMO—health maintenance organization, NSAID—non-steroidal anti-inflammatory drug, OA—osteoarthritis, SHAP—Shapley Additive exPlanations.

The top 15 predictors of potentially inappropriate NSAIDs use from the final XGBoost model are presented in Figure 3.

This figure displays both the feature importance and feature contribution to the model prediction. Each dot in the figure represents a single observation and low and high feature values are presented on a continuum with yellow representing low values and purple representing high values. As all categorical variables were converted into binary indicators, zero (i.e., absence) is indicated with yellow dots and one (i.e., presence) is represented by purple dots. The x-axis represents the marginal contribution of a feature to the change in the predicted probability of potentially inappropriate NSAIDs use. In terms of interpretation, for example, baseline non-selective NSAIDs use measured in days supplied is the most important predictor of potentially inappropriate use, and higher values (i.e., longer non-selective NSAID duration at baseline) are associated with a higher probability for potentially inappropriate NSAID use compared to lower values (i.e., shorter non-selective NSAID duration). For the categorical feature HMO, which ranked 9th in terms of feature importance, older adults enrolled in an HMO plan were less likely to have potentially inappropriate NSAIDs use as suggested by the larger spread of the purple dots on the left. For the CV and GI risk categories, high CV/low GI and low CV/high GI ranked 8th and 11th in terms of importance. Higher values for these variables (i.e., the presence of high CV/low GI or low CV/high GI) were associated with a higher probability of potentially inappropriate NSAIDs use, although not as large effect as non-selective or selective NSAID use duration variables. Other top predictors of potentially inappropriate NSAIDs use were: the number of selective NSAIDs use days, the FCI, age, the number of opioid prescriptions, physical therapy visits, or intra-articular corticosteroid injections, enrollment in an HMO plan, South region, female gender, other pain conditions, and hypertension (Figure 3).

**Figure 4:**
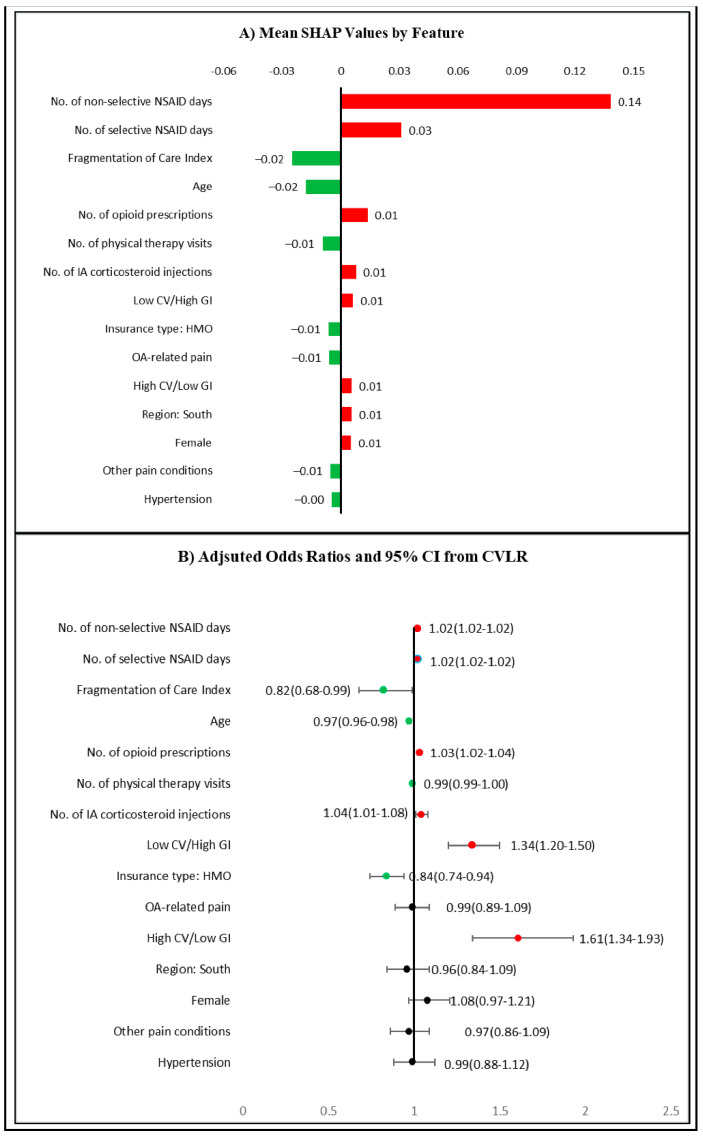
Association of Top Predictors to Potentially inappropriate NSAIDs. (**A**) Mean SHAP values by feature based on XGBoost. The bars show the average impact of a feature on XGBoost model output. (**B**) AOR and 95% CI for top 15 features based on an adjusted CVLR. The CVLR was adjusted for all features described in Section 2.4.2. Red bars/dots indicate an increase in the predicted probability/higher odds of potentially inappropriate NSAIDs use, respectively. Green bars/dots indicate a decrease in the predicted probability/lower odds of potentially inappropriate NSAIDs use, respectively. Based on 44,990 older adults (age > 65) with OA using data from Optum De-identified Clinformatics^®^ Data Mart who were continuously enrolled in Medicare Advantage plan during 2015–2016. AOR—adjusted odds ratio; CI—confidence interval, CV—cardiovascular, CVLR—cross-validated logistic regression, GI—gastrointestinal, HMO—health maintenance organization, NSAID—non-steroidal anti-inflammatory drug, OA—osteoarthritis, SHAP—Shapley Additive exPlanations, XGBoost—eXtreme Gradient Boosting.

The associations and average contributions of these features to the absolute predicted probability of potentially inappropriate NSAIDs use are presented in Figure 4A.

In this figure, red bars/dots indicate positive association and green bars/dots indicate negative associations of features with potentially inappropriate NSAIDs use, respectively. For example, the number of non-selective NSAIDs use days at baseline was the most important predictor and it increased the absolute values of the predicted probability of potentially inappropriate NSAIDs use at follow-up by an average of 0.14. Please note that the numbers presented in Figure 4A are average contributions for each feature to the model prediction. It is possible for individual values of any feature (e.g., 300 days of non-selective NSAID use) to have a probability higher or lower than that shown in the figure. As can be seen in Figure 4A, both the low CV/high GI and high CV/low GI risk categories were associated with a small but positive increase in the predicted probability of potentially inappropriate NSAIDs use at follow-up. Although age and the FCI were associated with lower predicted probabilities of potentially inappropriate NSAIDs use, Figure 3 highlights the presence of heterogeneity in prediction for these two variables. A closer examination of the distribution of SHAP values at different values of these variables (figure not shown) suggested that for similar values of FCI or age, the probability of potentially inappropriate NSAIDs use varied in both positive and negative directions.

Figure 4B presents results from the CVLR on potentially inappropriate NSAIDs use. Although the model was adjusted for all of the features mentioned above, results are presented only for the top 15 predictors identified by the XGBoost algorithm. The AORs indicate that a longer duration of non-selective or selective NSAIDs use were associated with higher odds of potentially inappropriate NSAIDs use (AOR: 1.02, 95% CI:1.02–1.02 for both). Similarly, the low CV/high GI (AOR: 1.34, 95% CI:1.20–1.50) and high CV/low GI risk (AOR: 1.61, 95% CI:1.34–1.93) categories were associated with higher odds of potentially inappropriate NSAIDs use. While most of the feature associations with potentially inappropriate NSAIDs use were similar to those derived from the XGBoost algorithm, some were different. For example, OA-related pain, South region, female gender, other pain conditions, and hypertension were not found to be significantly associated with potentially inappropriate NSAIDs use in CVLR. This could be due to the presence of nonlinear relationships of these variables to potentially inappropriate NSAIDs use.

## 4. Discussion

In our study cohort of older adults with OA, we observed that one in eight older adults had potentially inappropriate NSAIDs use. Specifically, we found that as many as 27% of older adults with OA had low CV/high GI risk for NSAIDs-related AEs, and 44.5% of these individuals were treated with potentially inappropriate NSAIDs for 60 days or longer. Moreover, despite old age being one of the risk factors for NSAIDs-related CV and GI AEs, we observed that NSAIDs use of 90 days or longer was present in 41.0% of individuals at low CV/low GI risk. Such NSAIDs use practices are in stark contrast with the clinical guidelines on long-term use of NSAIDs in older adults [23,37]. We also observed that long-term non-selective NSAIDs use was more common than selective NSAIDs use (8.5% vs. 1.9% with consecutive 90 days use) in our study cohort. This finding is alarming as previous research has shown that non-selective NSAIDs can increase the risk of both GI and CV AEs [8,9,38]. On the basis of our results, keeping a close watch on the duration of NSAIDs use in susceptible individuals may be warranted.

Our study findings of highly prevalent long-term NSAIDs use among those with existing CV and GI risk factors is concerning. This finding is in line with other published studies [18,19] which also reported that potentially inappropriate NSAIDs use is common in patients with OA. Although our study did not explore the reasons for NSAIDs prescriptions, a plausible reason could be the lack of alternative effective pain medications. A review of the comparative effectiveness of pain medications suggests that there is no “perfect” pain medication and NSAIDs may be the safer option available to both patients and physicians [39]. With the opioid epidemic in the USA and resulting stricter controls on their use, physicians and patients may rely on NSAIDs for pain relief despite adverse consequences. As NSAIDs will continue to hold an important place in the clinical management of pain for OA patients, our findings suggest a need for heightened surveillance of older adults with OA and co-existing CV and GI conditions. Clinicians may benefit from using tools like the GI risk score tool to identify patients at high risk for GI AEs and to individualize their treatment plan [38]. A drug utilization review system and computerized decision support tools may also help to identify such patients and make changes to their treatment as necessary.

We also found that the baseline NSAIDs use irrespective of the type (COX-2 selective or non-selective) was the leading predictor of potentially inappropriate NSAIDs use during follow-up. This finding is consistent with another published study on high-risk primary care patients which reported that prior NSAIDs use was strongly associated with current use [40]. While we did not study the reasons behind this association, there are several factors that may have played a role. One of these factors could be the lack of knowledge in NSAID users about their AEs and contraindications. In a study of over-the-counter (OTC) NSAID users surveyed at a rheumatology clinic, one-third were unaware of their contraindications [41]. Similar reports of the lack of patient knowledge about NSAIDs-related AEs have been published as well [42,43]. Keeping these findings in mind, it is important that patients taking NSAIDs are better informed about the possible AEs. The other factor could be the formulary restrictions on the use of selective NSAID use. A study by Louder et al. reported that formulary restrictions for celecoxib among Medicare beneficiaries resulted in lower rates of use of celecoxib (the only available selective NSAIDs in the US) and higher rates of use for non-selective NSAIDs [44]. The study also reported that the incidence and costs associated with GI AEs were higher for individuals with formulary restrictions [44]. While formulary restrictions can reduce overutilization of expensive medicines, they can also increase the burden on patients and prescribers making it difficult for them to access the restricted medicine when necessary [45].

We also observed that older adults with visits to multiple providers, i.e., higher FCI, had a lower probability of potentially inappropriate NSAIDs use. This finding could be explained by at least two possible reasons. First, individuals with more encounters with the health care system may have a higher likelihood of their potentially inappropriate NSAIDs use being identified by a health care worker. Second, individuals with higher FCI may represent a subset of patients with higher disease severity and/or dissatisfaction with their current treatment. This may further reduce the chances of a person being treated with NSAIDs for long. Additionally, we observed that there was a considerable variation in the predicted probability of potentially inappropriate NSAID use for individuals with the same FCI values. Further research into what factors related to care fragmentation lead to an increase in the probability of potentially inappropriate NSAIDs use is needed. On the basis of the association between the FCI and potentially inappropriate NSAID use, it needs to be seen whether frequent follow-ups of patients on long-term NSAID therapy can help reduce the risk of potentially inappropriate NSAID use.

In our study, we observed that patients using other pain medications such as opioids and intra-articular corticosteroids at baseline had a higher probability of potentially inappropriate NSAIDs use. The combined use of multiple classes of pain medications is common among adults with OA or those with chronic pain and has been increasing over time [46,47]. Studies have shown that the combined use of opioids and NSAIDs may provide effective pain relief in 90% of adults with chronic pain [48], despite increased risk of AEs associated with such use. It is possible that older adults with OA are prescribed multiple pain medications with different mechanisms of action to achieve a synergistic effect. Patients with moderate or severe pain have been shown to regularly use combination analgesics which may explain the higher likelihood of such patients receiving potentially inappropriate NSAIDs [49].

The higher number of physical therapy visits during the baseline period were associated with a lower probability of potentially inappropriate NSAIDs use, suggesting that non-pharmacological interventions may need to be considered in high CV and GI risk patients to reduce their risk of NSAIDs-related AEs. Physicians may also consider a multimodal approach that combines pharmacological and non-pharmacological treatments for pain relief in OA adults. An alternative explanation could be that older OA patients using physical therapy may be more physically active and/or may have less severe OA requiring shorter or no treatment with NSAIDs.

In summary, the results from our study suggest a need for heightened surveillance for CV and GI risk factors in older adults with OA and CV and GI risk factors requiring treatment with NSAIDs. Older adults who have multiple risk factors (e.g., females with baseline long-term NSAIDs use and high CV or GI risk) that increase the probability of potentially inappropriate NSAIDs use should be watched closely for their NSAIDs use, including the OTC drugs. The advent of new therapies with the ability to target the underlying pathophysiology of OA may help reduce the reliance of patients on NSAIDs against recommendations [50].

The limitations of our study include its retrospective and observational nature; the possible underestimation of clinical conditions in claims data; not capturing patients who do not routinely see clinicians or who do not use health services; lack of information on variables that can affect NSAIDs use (e.g., patients’ dietary and personal health habits, OTC NSAIDs use including low-dose aspirin, OA severity, pain, and patients’ response to pain treatment); the possibility of a filled NSAID prescriptions not being used by the patient; and lack of generalizability to all older adults in the USA. Despite these limitations, our study has many strengths. It is the first study to document the rates of potentially inappropriate NSAIDs use in a cohort of older adults with OA. It is also the first study to examine the prevalence of combinations of CV and GI risk factors and employ machine learning algorithms with a comprehensive list of 31 features to identify leading predictors and direction of their associations with potentially inappropriate NSAIDs use.

## 5. Conclusions

Our findings suggest that one in eight older adults with OA received prescriptions for NSAIDs that were not in line with their CV and GI risk profile. Our results showed that patients with high GI or high CV risk were more likely to receive potentially inappropriate NSAIDs. Our study also demonstrated that machine learning techniques can be used to identify the predictors of potentially inappropriate NSAIDs use using real-world data. Some of the leading predictors of potentially inappropriate NSAID use were modifiable such as the length of NSAIDs, FCI, use of other pain medications, and the presence of other pain conditions. Such information can be used to influence policy, clinical practice, and patient education. Future studies with a prospective cohort design to explore the reasons for long-term NSAIDs use in individuals with high CV and GI risk profile are warranted to inform targeted interventions for reducing NSAIDs-related AEs.

## Figures and Tables

**Table 1:** Identification of High Risk Older Adults [24,25,30].

Gastrointestinal Risk Factors (at Least One of the Following)	Cardiovascular Risk Factors (at Least One of the Following)
Complicated peptic ulcer disease	Angina
Uncomplicated peptic ulcer disease	Stroke
Dyspepsia	Myocardial infarction
Gastroesophageal reflux disorder	Congestive heart failure
Concomitant use of one of the following drugs with NSAIDs ^a^: Corticosteroids Antiplatelets Selective serotonin reuptake inhibitors Aspirin ^b^ Anticoagulants	

See supplementary materials for ICD-10 codes used to identify these conditions. NSAIDs- non-steroidal anti-inflammatory drugs. ^a^ Concomitant use of at least 7 consecutive days was required. ^b^ Over-the-counter aspirin use was not captured.

**Table 2:** Selected Sample Characteristics; Older Adults (age > 65) with Osteoarthritis (*n* = 44,990); Optum Clinformatics Data Mart 2015–2016.

Variable		N/Mean	Percentage/SD
All		44,990	100.0
Age			
	65–69	10,315	22.9
	70–74	11,330	25.2
	75–79	8957	19.9
	80+	14,388	32.0
Sex			
	Female	29,853	66.4
	Male	15,137	33.6
Region			
	Northeast	6034	13.4
	Midwest	10,862	24.1
	South	16,410	36.5
	West	11,684	26.0
Insurance Type			
	HMO	16,314	36.3
	Other	28,676	63.7
Other Pain Conditions			
	Yes	15,733	35.0
	No	29,257	65.0
OA-related pain			
	Yes	24,880	55.3
	No	20,110	44.7
Anxiety			
	Yes	6285	14.0
	No	38,705	86.0
Substance abuse disorder			
	Yes	3354	7.5
	No	41,636	92.5
Depression			
	Yes	7620	16.9
	No	37,370	83.1
Fragmentation of care index		0.44	0.28
Number of IA hyaluronic acid injections			
		0.2	0.9
Number of IA corticosteroid injections			
		0.8	1.5
Number of physical therapy visits			
		4.4	12.0
Number of opioid prescriptions			
		2.0	4.2
Any NSAIDs use			
	Yes	11,891	21.6
	No	33,099	78.4
CV/GI Risk			
	High CV/high GI	3383	7.5
	High CV/low GI	4233	9.4
	Low CV/high GI	12,145	27.0
	Low CV/low GI	25,229	56.1
Number of days of non-selective NSAIDs use			
		29.3	79.5
Number of days of selective NSAIDs use			
		5.8	38.7
Potentially inappropriate NSAIDs use at follow-up			
	Yes	5772	12.8
	No	39,218	87.2

Note: Based on 44,990 older adults (age > 65) with OA using data from Optum’s De-identified Clinformatics^®^ Data Mart who were continuously enrolled in Medicare Advantage plan during 2015–2016. All characteristics were captured at baseline unless specified. CV—cardiovascular; GI—gastrointestinal; HMO, health maintenance organization; IA, intra-articular; NSAIDs—non-steroidal anti-inflammatory drugs; OA—osteoarthritis.

**Table 3:** Model Performance Metrics Using Test data *; Potentially Inappropriate NSAIDs Use in Older Adults with Osteoarthritis; Optum Clinformatics Data Mart 2015–2016.

	XGBoost	Cross-Validated Logistic Regression
Accuracy	0.93	0.93
Precision	0.81	0.83
Recall	0.63	0.58
F1 Score	0.71	0.68
Specificity	0.98	0.98
Kappa	0.67	0.64
AUROC	0.92	0.91

Note: Based on 44,990 older adults (age > 65) with OA using data from Optum De-identified Clinformatics^®^ Data Mart who were continuously enrolled in Medicare Advantage plan during 2015–2016. * Test data consisted of a 30% random sample of the original dataset.

## Data Availability

Restrictions apply to the availability of Optum De-identified Clinformatics^®^ Data Mart data. Data was obtained from Optum® through third-party license and authors cannot make these data publicly available dur to data use agreement.

## Supplementary Materials

The following are available online at https://www.mdpi.com/1660-4601/18/1/155/s1. Table S1. ICD-10 Diagnosis codes for gastrointestinal and cardiovascular risk factors.

## References

[1] Zhou Y., Boudreau D.M., Freedman A.N. (2014). Trends in the Use of Aspirin and Nonsteroidal Anti-Inflammatory Drugs in the General U.S. Population. Pharmacoepidemiol. Drug Saf..

[2] Cooper C., Chapurlat R., Al-Daghri N., Herrero-Beaumont G., Bruyère O., Rannou F., Roth R., Uebelhart D., Reginster J.-Y. (2019). Safety of Oral Non-Selective Non-Steroidal Anti-Inflammatory Drugs in Osteoarthritis: What Does the Literature Say?. Drugs Aging.

[3] Kolasinski S.L., Neogi T., Hochberg M.C., Oatis C., Guyatt G., Block J., Callahan L., Copenhaver C., Dodge C., Felson D. (2020). 2019 American College of Rheumatology/Arthritis Foundation Guideline for the Management of Osteoarthritis of the Hand, Hip, and Knee. Arthritis Care Res..

[4] Bhala N., Emberson J., Merhi A., Abramson S., Arber N., Baron J.A., Bombardier C., Cannon C., Farkouh M.E., Coxib and traditional NSAID Trialists’ (CNT) Collaboration (2013). Vascular and Upper Gastrointestinal Effects of Non-Steroidal Anti-Inflammatory Drugs: Meta-Analyses of Individual Participant Data from Randomised Trials. Lancet.

[5] Rahme E., Joseph L., Kong S.X., Watson D.J., LeLorier J. (2000). Gastrointestinal Health Care Resource Use and Costs Associated with Nonsteroidal Antiinflammatory Drugs versus Acetaminophen: Retrospective Cohort Study of an Elderly Population. Arthritis Rheum..

[6] Bidaut-Russell M., Gabriel S.E. (2001). Adverse Gastrointestinal Effects of NSAIDs: Consequences and Costs. Best Pract. Res. Clin. Gastroenterol..

[7] (2019). National Institute for Health and Care Excellence. NSAIDs—Prescribing Issues.

[8] Helin-Salmivaara A., Virtanen A., Vesalainen R., Grönroos J.M., Klaukka T., Idänpään-Heikkilä J.E., Huupponen R. (2006). NSAID Use and the Risk of Hospitalization for First Myocardial Infarction in the General Population: A Nationwide Case-Control Study from Finland. Eur. Heart J..

[9] Osani M.C., Vaysbrot E.E., Zhou M., McAlindon T.E., Bannuru R.R. (2020). Duration of Symptom Relief and Early Trajectory of Adverse Events for Oral NSAID s in Knee Osteoarthritis: A Systematic Review and Meta-analysis. Arthritis Care Res..

[10] Center for Drug Evaluation and Research FDA Drug Safety Communication: FDA Strengthens Warning That Non-Aspirin Nonsteroidal Anti-Inflammatory Drugs (NSAIDs) Can Cause Heart Attacks or Strokes. http://www.fda.gov/drugs/drug-safety-and-availability/fda-drug-safety-communication-fda-strengthens-warning-non-aspirin-nonsteroidal-anti-inflammatory.

[11] Zhang Y., Jordan J.M. (2010). Epidemiology of Osteoarthritis. Clin. Geriatr. Med..

[12] Hawker G.A., Gignac M.A.M., Badley E., Davis A.M., French M.R., Li Y., Perruccio A.V., Power J.D., Sale J., Lou W. (2011). A Longitudinal Study to Explain the Pain-Depression Link in Older Adults with Osteoarthritis. Arthritis Care Res. (Hoboken).

[13] Zhao X., Shah D., Gandhi K., Wei W., Dwibedi N., Webster L.R., Sambamoorthi U. (2019). Clinical, Humanistic, and Economic Burden of Osteoarthritis among Noninstitutionalized Adults in the United States. Osteoarthr. Cartil..

[14] (2009). American Geriatrics Society Panel on Pharmacological Management of Persistent Pain in Older Persons Pharmacological Management of Persistent Pain in Older Persons. J. Am. Geriatr. Soc..

[15] Griffin M.R. (1998). Epidemiology of Nonsteroidal Anti-Inflammatory Drug-Associated Gastrointestinal Injury. Am. J. Med..

[16] Wegman A., van der Windt D., van Tulder M., Stalman W., de Vries T. (2004). Nonsteroidal Antiinflammatory Drugs or Acetaminophen for Osteoarthritis of the Hip or Knee? A Systematic Review of Evidence and Guidelines. J. Rheumatol..

[17] Vanderstraeten G., Lejeune T.M., Piessevaux H., De Bacquer D., Walker C., De Beleyr B. (2016). Gastrointestinal Risk Assessment in Patients Requiring Non-Steroidal Anti-Inflammatory Drugs for Osteoarthritis: The GIRANO Study. J. Rehabil. Med..

[18] Lanas A., Garcia-Tell G., Armada B., Oteo-Alvaro A. (2011). Prescription Patterns and Appropriateness of NSAID Therapy According to Gastrointestinal Risk and Cardiovascular History in Patients with Diagnoses of Osteoarthritis. BMC Med..

[19] Sebaldt R.J., Petrie A., Goldsmith C.H., Marentette M.A. (2004). Appropriateness of NSAID and Coxib Prescribing for Patients with Osteoarthritis by Primary Care Physicians in Ontario: Results from the CANOAR Study. Am. J. Manag. Care.

[20] Optum Clinformatics Data Mart. https://www.optum.com/content/dam/optum/resources/productSheets/Clinformatics_for_Data_Mart.pdf.

[21] Shrestha S., Dave A.J., Losina E., Katz J.N. (2016). Diagnostic Accuracy of Administrative Data Algorithms in the Diagnosis of Osteoarthritis: A Systematic Review. BMC Med. Inform. Decis. Mak..

[22] Chan F.K.L., Abraham N.S., Scheiman J.M., Laine L. (2008). First International Working Party on Gastrointestinal and Cardiovascular Effects of Nonsteroidal Anti-inflammatory Drugs and Anti-platelet Agents Management of Patients on Nonsteroidal Anti-Inflammatory Drugs: A Clinical Practice Recommendation from the First International Working Party on Gastrointestinal and Cardiovascular Effects of Nonsteroidal Anti-Inflammatory Drugs and Anti-Platelet Agents. Am. J. Gastroenterol..

[23] Hochberg M.C., Altman R.D., April K.T., Benkhalti M., Guyatt G., McGowan J., Towheed T., Welch V., Wells G., Tugwell P. (2012). American College of Rheumatology 2012 Recommendations for the Use of Nonpharmacologic and Pharmacologic Therapies in Osteoarthritis of the Hand, Hip, and Knee. Arthritis Care Res. (Hoboken).

[24] Lanas A., Tornero J., Zamorano J.L. (2010). Assessment of Gastrointestinal and Cardiovascular Risk in Patients with Osteoarthritis Who Require NSAIDs: The LOGICA Study. Ann. Rheum. Dis..

[25] Boyapati R., Ong S.Y., Ye B., Kruavit A., Lee N., Vaughan R., Nandurkar S., Gibson P., Garg M. (2014). One Fifth of Hospitalizations for Peptic Ulcer-Related Bleeding Are Potentially Preventable. World J. Gastroenterol..

[26] Lanza F.L., Chan F.K.L., Quigley E.M.M. (2009). Practice Parameters Committee of the American College of Gastroenterology Guidelines for Prevention of NSAID-Related Ulcer Complications. Am. J. Gastroenterol..

[27] Bhatt D.L., Scheiman J., Abraham N.S., Antman E.M., Chan F.K.L., Furberg C.D., Johnson D.A., Mahaffey K.W., Quigley E.M. (2008). American College of Cardiology Foundation Task Force on Clinical Expert Consensus Documents ACCF/ACG/AHA 2008 Expert Consensus Document on Reducing the Gastrointestinal Risks of Antiplatelet Therapy and NSAID Use: A Report of the American College of Cardiology Foundation Task Force on Clinical Expert Consensus Documents. Circulation.

[28] García Rodríguez L.A., Jick H. (1994). Risk of Upper Gastrointestinal Bleeding and Perforation Associated with Individual Non-Steroidal Anti-Inflammatory Drugs. Lancet.

[29] Gabriel S.E., Jaakkimainen L., Bombardier C. (1991). Risk for Serious Gastrointestinal Complications Related to Use of Nonsteroidal Anti-Inflammatory Drugs. A Meta-Analysis. Ann. Intern. Med..

[30] Aday L.A., Andersen R. (1974). A Framework for the Study of Access to Medical Care. Health Serv Res.

[31] Liu C.W., Einstadter D., Cebul R.D. (2010). Care Fragmentation and Emergency Department Use among Complex Patients with Diabetes. Am. J. Manag. Care.

[32] Boulesteix A.-L., Schmid M. (2014). Machine Learning versus Statistical Modeling. Biom. J..

[33] Borstelmann S.M. (2020). Machine Learning Principles for Radiology Investigators. Acad. Radiol..

[34] Chen T., Guestrin C. (2016). XGBoost: A Scalable Tree Boosting System. Proceedings of the 22nd ACM SIGKDD International Conference on Knowledge Discovery and Data Mining.

[35] Zhang Z., Ho K.M., Hong Y. (2019). Machine Learning for the Prediction of Volume Responsiveness in Patients with Oliguric Acute Kidney Injury in Critical Care. Crit. Care.

[36] Lundberg S.M., Lee S.-I., Guyon I., Luxburg U.V., Bengio S., Wallach H., Fergus R., Vishwanathan S., Garnett R. (2017). A Unified Approach to Interpreting Model Predictions. Proceedings of the Advances in Neural Information Processing Systems 30.

[37] By the 2019 American Geriatrics Society Beers Criteria® Update Expert Panel (2019). American Geriatrics Society 2019 Updated AGS Beers Criteria® for Potentially Inappropriate Medication Use in Older Adults: 2019 AGS BEERS CRITERIA® UPDATE EXPERT PANEL. J. Am. Geriatr. Soc..

[38] Fries J.F., Williams C.A., Bloch D.A., Michel B.A. (1991). Nonsteroidal Anti-Inflammatory Drug-Associated Gastropathy: Incidence and Risk Factor Models. Am. J. Med..

[39] Smith S.R., Deshpande B.R., Collins J.E., Katz J.N., Losina E. (2016). Comparative Pain Reduction of Oral Non-Steroidal Anti-Inflammatory Drugs and Opioids for Knee Osteoarthritis: Systematic Analytic Review. Osteoarthr. Cartil..

[40] Bouck Z., Mecredy G.C., Ivers N.M., Barua M., Martin D., Austin P.C., Tepper J., Bhatia R.S. (2018). Frequency and Associations of Prescription Nonsteroidal Anti-Inflammatory Drug Use Among Patients With a Musculoskeletal Disorder and Hypertension, Heart Failure, or Chronic Kidney Disease. JAMA Intern. Med..

[41] Arain A., Rasheed M., Sallam N., Sarwar Z., Khan M. (2019). Patient’s Knowledge and Use of Oral Non-Steroidal Anti-Inflammatory Drugs in a Rheumatology Clinic. Kans. J. Med..

[42] Wynne H.A., Long A. (1996). Patient Awareness of the Adverse Effects of Non-Steroidal Anti-Inflammatory Drugs (NSAIDs). Br. J. Clin. Pharmacol..

[43] Gibson J.C. (2020). A Survey of Patients Knowledge of NSAID Side Effects. BMJ.

[44] Louder A.M., Joshi A.V., Ball A.T., Cappelleri J.C., Deminski M.C., Sanchez R.J. (2011). Impact of Celecoxib Restrictions in Medicare Beneficiaries with Arthritis. Am. J. Manag. Care.

[45] Carlton R.I., Bramley T.J., Nightengale B., Conner T.M., Zacker C. (2010). Review of Outcomes Associated With Formulary Restrictions: Focus on Step Therapy. Am. J. Pharm. Benefits.

[46] Kingsbury S.R., Hensor E.M., Walsh C.A., Hochberg M.C., Conaghan P.G. (2013). How Do People with Knee Osteoarthritis Use Osteoarthritis Pain Medications and Does This Change over Time? Data from the Osteoarthritis Initiative. Arthritis Res. Ther..

[47] Marcum Z.A., Perera S., Donohue J.M., Boudreau R.M., Newman A.B., Ruby C.M., Studenski S.A., Kwoh C.K., Simonsick E.M., Bauer D.C. (2011). Analgesic Use for Knee and Hip Osteoarthritis in Community-Dwelling Elders. Pain Med..

[48] Slater D., Kunnathil S., McBride J., Koppala R. (2010). Pharmacology of Nonsteroidal Antiinflammatory Drugs and Opioids. Semin. Intervent. Radiol..

[49] Blamey R., Jolly K., Greenfield S., Jobanputra P. (2009). Patterns of Analgesic Use, Pain and Self-Efficacy: A Cross-Sectional Study of Patients Attending a Hospital Rheumatology Clinic. BMC Musculoskelet. Disord..

[50] Ghouri A., Conaghan P.G. (2019). Update on Novel Pharmacological Therapies for Osteoarthritis. Ther. Adv. Musculoskelet. Dis..

