# Supplementary material for: A Machine Learning Approach to Identify Predictors of Potentially Inappropriate Non-Steroidal Anti-Inflammatory Drugs (NSAIDs) Use in Older Adults with Osteoarthritis
Source: Int J Environ Res Public Health. 2020 Dec 28;18(1):155. doi: 10.3390/ijerph18010155 (PMC7794853; doi:10.3390/ijerph18010155)
Supplement: Supplementary file 1 [file ijerph-18-00155-s001.pdf]

Table S1. ICD-10 Diagnosis codes for gastrointestinal and cardiovascular risk factors.

**Disease or Condition**	**ICD-10 codes**
Complicated peptic ulcer disease[25,56]	K25.0-K25.6, K26.1-K26.2, K26.4-K26.6, K27.1-27.2, K27.4-K27.6, K28.1-28.2, K28.4-K28.6, K29.01, K29.21, K29.41, K29.51, K29.61, K29.71, K29.81, K29.91, K92.0-92.2
Uncomplicated peptic ulcer disease [25,56]	K25.3, K25.7, K25.9, K26.3, K26.7, K26.9, K27.3, K27.7, K27.9, K28.3, K28.7, K28.9, Z87.11
Dyspepsia [57]	K30
Gastroesophageal reflux disorder [57]	K21.0, K21.9
Angina [58]	I20.0, I20.1, I20.8, I20.9
Stroke [59]	I60.x, I61.x, I63.x, I64.x, H34.1, G45.x
Myocardial infarction [57]	I21.09, I21.11, I21.19, I21.3, I21.4, I21.9, I21.A9, I25.2
Congestive heart failure [60]	I50.xx
